# Direct oral anticoagulants in treatment of cerebral venous thrombosis: a systematic review protocol

**DOI:** 10.1186/s13643-019-1022-8

**Published:** 2019-04-18

**Authors:** Gauruv Bose, Justin Graveline, Vignan Yogendrakumar, Dean Fergusson, Dar Dowlatshahi

**Affiliations:** Department of Medicine, University of Ottawa and The Ottawa Hospital Research Institute, 1053 Carling Avenue, Ottawa, ON K1Y 4E9 Canada

## Abstract

**Background and purpose:**

Cerebral venous thrombosis causes disability from venous infarct and hemorrhage and potential mortality. Anticoagulation improves survival and disability outcomes, yet direct oral anticoagulants are currently not indicated in cerebral venous thrombosis due to lack of evidence, despite being on the market for nearly a decade. This systematic review will collate evidence of reported safety and efficacy of direct oral anticoagulant therapy in cerebral venous thrombosis.

**Methods:**

A search strategy was developed with a research librarian and registered on a protocol database (PROSPERO CRD42017078398). All published studies from MEDLINE and EMBASE up to February 2019 containing patients diagnosed with cerebral venous thrombosis who were treated with a direct oral anticoagulant (dabigatran, rivaroxaban, apixaban, or edoxaban) will be included. A risk of bias analysis will be performed to evaluate quality of studies overall.

**Discussion:**

Current guidelines in the treatment of cerebral vein thrombosis dating back to 2011 from the American Heart Association/American Stroke Association endorse the utility of anticoagulation for the treatment of cerebral vein thrombosis; however, they did not support the use of direct oral anticoagulants. Updated guidelines from the European Stroke Organization, endorsed by the European Academy of Neurology in 2017, also refute utilization of direct oral anticoagulants due to a lack of evidence. There have been nearly 10 years of experience with direct oral anticoagulants in the treatment of venous thrombosis and prevention of stroke in patients with atrial fibrillation, with purported efficacy and safety in comparison with heparins and vitamin K antagonists. Our goal is to undertake a systematic review to assess the effectiveness and safety of direct oral anticoagulants in patients with cerebral vein thrombosis to help guide clinical decision-making for patients unable to take heparins or vitamin K antagonists and to direct future studies to contribute further to an area of certain evidence-based needs.

**Systematic review registration:**

PROSPERO CRD42017078398

**Electronic supplementary material:**

The online version of this article (10.1186/s13643-019-1022-8) contains supplementary material, which is available to authorized users.

## Background

### Rationale

Cerebral vein thrombosis (CVT) is a medical emergency requiring rapid treatment to prevent venous infarct, hemorrhage, and eventual neurologic disability or mortality. The incidence is estimated at 1 per 100,000 per year with mean age of 39 years, and although the mortality rate has reduced from 15 to 5% over the past 50 years due to advances in treatment and detection, morbidity rates can reach as high as 20–30% thus necessitating quick and adequate treatment [1]. Treatment with anticoagulation for cerebral venous thrombosis (CVT) was first shown to be beneficial in a prospective study in 1991 by Einhäupl et al. disputing previous concerns for increasing intracranial hemorrhage risk [2]. This finding has been replicated, and a 2011 systematic review by Coutinho et al. supports international guidelines for acute treatment of CVT with heparin followed by longer-term oral vitamin K antagonist (VKA) [3–5].

The introduction of direct oral anticoagulants (DOAC) in the early 2010s allowed for a viable alternative to VKA or heparins for treatment of venous thrombosis and to prevent stroke in patients with atrial fibrillation in both the acute and longer-term phases. The DOAC demonstrated increased utility compared with VKA by more predictable pharmacokinetics not requiring international normalized ratio (INR) testing or complicated dose adjustments, while at the same time having similar efficacy to VKA in treatment of acute venous thromboembolism (VTE), similar, if not better, rates of bleeding and mortality [6, 7]. Subsequently, increased prescribing of these medications has been seen in the last decade. However, due to the rarity of CVT, trials comparing DOAC to heparin or VKA have been minimal, as such, the published guidelines recommend not using DOAC in treatment of CVT despite the potential advantages.

### Objective

This systematic review will collate evidence of reported safety and efficacy of direct oral anticoagulant therapy in cerebral venous thrombosis.

## Methods

### Eligibility criteria

The eligible population includes patients of any age with a diagnosis of cerebral vein thrombosis (by cerebral angiogram, magnetic resonance venography (MRV), or computed tomographic venography (CTV)) who were treated with a direct oral anticoagulant (apixaban, rivaroxaban, dabigatran, or edoxaban) at any point of treatment, the acute or long-term phase. All studies with or without a comparison group are eligible for this review, including prospective trials or retrospective cohorts, case reports, or case series. No comparison group is required for studies, and reported outcomes are expected to vary in primarily retrospective or case reports, so specific outcome measures are not explicitly required for eligibility. See the “Data items, outcomes, and prioritization” section for extracted data. Studies to be included may be in any language.

### Information sources

Included sources will be Cochrane Stroke Group Trials Register (last searched on February 2019), Ovid MEDLINE, Epub Ahead of Print, In-Process & Other Non-Indexed Citations, Ovid MEDLINE(R) Daily and Ovid MEDLINE(R) (1946 to February 2019), EMBASE (1947 to February 2019), and the Cochrane Central Register of Controlled Trials (1950 to February 2019). Where applicable, authors of abstracts will be contacted for further data as well as reference check for published reviews.

### Protocol and registration

A search strategy was developed with a research librarian. The design of this systematic review follows the PRISMA-P [8] and PRISMA [9] guidelines where applicable. The protocol is hosted on the registered database, PROSPERO (CRD42017078398) [10]. See Additional file 1 for the search strategy and Additional file 2 for the PRISMA-P checklist.

### Study records

#### Data management and collection

Two authors (GB, JG) will independently extract data capturing pertinent demographics, treatment dosage and duration, and follow-up and outcomes. For incomplete data, authors will be contacted for any possible missing data. A single reviewer (GB) will aggregate these study details into an Excel spreadsheet for analysis.

#### Selection process

Two independent reviewers will select included studies in a two-stage screening process. In stage one, two authors (GB, JG) screen abstracts and titles for potentially relevant articles, and in stage two, two authors (GB, JG) read potentially relevant articles to confirm that they met the inclusion criteria. At the end of each stage, disagreements will be resolved by a third reviewer (DD).

#### Data items, outcomes, and prioritization

Data items were selected based on the research objective and questions stated above. We will extract the following: general study information including date, country, language, number of patients, and type of study; participant characteristics including age, sex, medical history, medications, symptoms, and examination findings; information on CVT diagnosis including imaging modality, location of venous thrombosis, and other imaging findings such as edema or intracranial hemorrhage; and intervention details including DOAC name, dosage, and length of treatment.

Outcome data will be categorized into safety and efficacy data. Safety outcome results are primary outcomes given that DOAC therapy is off-label use in CVT, so emphasis of a safety signal is the primary goal. Safety data includes occurrence of intracranial bleeding, extracranial bleeding, mortality, and any other reported adverse events. The secondary outcomes are efficacy data including recanalization time and rates, the initial and final modified Rankin Scale, and the initial and final National Institute of Health Stroke Scale score.

#### Risk of bias assessment of individual studies

The expected majority of articles are either case report or case series given the rarity of this disease entity and novelty of DOAC use in treating CVT, therefore two risk of bias tools will be employed for careful assessment of potential bias. The two tools include ROBINS-I [11] and the Joanna Briggs Institute Critical Appraisal Checklists [12].

#### Data synthesis

The purpose of this systematic review is to explore the evidence of DOAC use in CVT to assess for safety and efficacy, as well as guide protocol design for future prospective studies. The current guidelines state that DOAC use is not indicated in CVT because no high-level studies have been done as of yet; however, multiple case reports and case series have been published in the literature which have not been systematically reviewed, as well as possibly small trials. Our goal for data synthesis will be to qualitatively and descriptively present the data, and if possible, inferential statistics and meta-analysis will be performed.

## Discussion

This systematic review will investigate the experience DOAC use in CVT, with assessment of reported safety and efficacy. The benefits of DOAC in comparison with VKA include more predictable pharmacokinetics resulting in no necessity of blood work checking of international normalized ratio (INR) and fewer interactions with other medications or foods, as well as reduced risk of intracranial hemorrhage [7]. The population suffering from CVT has a reported morbidity from intracranial hemorrhage or venous infarct as high as 20–30%, and the mean age is 39 years, thus investigation and utilization of potentially advantageous medications are certainly needed [1]. It is understandable that shortly after the introduction of DOAC, first with dabigatran in 2009, the guidelines published by the American Heart Association/American Stroke Association had insufficient evidence to recommend DOAC use; although after nearly a decade, it is concerning that not much has changed on the evidence front [4, 6].

A recently reported guideline has been published by the European Stroke Organization in 2017, strongly recommended acute treatment with therapeutic heparin based on two randomized trials, however stated that there is still insufficient evidence to use DOAC in CVT based on two published case series and not by systematic review [5]. The two studies were seven patients treated with rivaroxaban, published in 2014 [13], and 15 patients treated with dabigatran published in 2015 [14]. Given this statement, we are expecting the available studies to be of generally low-quality observational cohorts and vulnerable to bias. This is important to systematically investigate and report, since any future interventional studies reporting DOAC therapy in CVT would need to be designed to avoid such bias.

An argument to conduct prospective studies of DOAC use in CVT has been made over the past decade. Two prospective studies comparing rivaroxaban to VKA have been begun protocol development but have not yet started recruitment: “Study of Rivaroxaban for CeREbral Venous Thrombosis (SECRET)” (ClinicalTrials.gov Identifier NCT03178864) [15] and “Comparison of the efficacy of rivroxaban to Coumadin (warfarin) in cerebral venous thrombosis (CVT)” (ClinicalTrials.gov Identifier NCT03191305) [16]. One prospective study comparing dabigatran to VKA has begun recruitment: “The efficacy and safety of dabigatran etexilate for the treatment of cerebral venous thrombosis” (ClinicalTrials.gov Identifier NCT03217448) [17], and one study has completed recruitment and reported preliminary results at an international conference: “A clinical trial comparing efficacy and safety of dabigatran etexilate with warfarin in patients with cerebral venous and dural sinus thrombosis (RE-SPECT CVT)” (ClinicalTrials.gov Identifier NCT02913326), we aim to include the results of this study in our analysis once published [18, 19]. Given the rarity of CVT, recruitment of patients to these studies has been slow.

Performing a systematic review at this point is important since DOAC use in CVT is gaining attention in upcoming trials; however, due to slow recruitment, guidelines are not expected to change for some time. This review will serve to summarize available safety and efficacy evidence, as well as highlight potential bias. The review may also indicate how patient outcomes and treatment details are reported in order to assist future trial design as well as reporting of retrospective cases. If the review uncovers significant safety or efficacy concerns in the current literature, they will be flagged for prospective studies to prove or dispute such signals.

## Additional files

Additional file 1: Search strategy. (DOCX 15 kb)

Additional file 2: PRISMA-P checklist [8]. (DOCX 22 kb)

## Abbreviations

CTV: Computed tomographic venography
CVT: Cerebral vein thrombosis
DOAC: Direct oral anticoagulant
INR: International normalized ratio
MRV: Magnetic resonance venography
PRISMA: Preferred Reporting Items for Systematic Reviews and Meta-Analyses
PRISMA-P: Preferred Reporting Items for Systematic Reviews and Meta-Analyses Protocols
PROSPERO: International Prospective Register of Systematic Reviews
ROBINS-I: Risk Of Bias In Non-Randomized Studies - of Interventions
VKA: Vitamin K antagonist
VTE: Venous thromboembolism
