# Supplementary material for: Direct oral anticoagulants in treatment of cerebral venous thrombosis: a systematic review protocol
Source: Syst Rev. 2019 Apr 18;8:99. doi: 10.1186/s13643-019-1022-8 (PMC6474061; doi:10.1186/s13643-019-1022-8)
Supplement: Supplementary file 1 — Search strategy. (DOCX 15 kb) [file 13643_2019_1022_MOESM1_ESM.docx]

**Additional file 1: Search Strategy**

**Search from** Embase Classic+Embase <1947 to present>, EBM Reviews - Cochrane Central Register of Controlled Trials <Present>, Ovid MEDLINE(R) Epub Ahead of Print, In-Process & Other Non-Indexed Citations, Ovid MEDLINE(R) Daily and Ovid MEDLINE(R) <1946 to present> :

- 1  apixaban.mp. (10426)
- 2  edoxaban.mp. (3890)
- 3  Dabigatran.mp. (16687)
- 4  Rivaroxaban.mp. (16154)
- 5  (doac* or noac*).tw,kw. (6527)
- 6  ((direct oral or novel) adj3 (anticoagul* or anti coagulat*)).tw. (7212)
- 7  exp Factor Xa Inhibitors/ (25058)
- 8  Factor Xa Inhibit*.mp. (8883)
- 9  Antithrombins/ or thrombin inhibit*.mp. (29198)
- 10  or/1-9 (63305)
- 11  "intracranial embolism and thrombosis"/ or intracranial thrombosis/ or exp sinus thrombosis,

intracranial/ (86245)

- 12  cvt.tw,kw. (3544)
- 13  (cerebral veins/ or exp cranial sinuses/) and (thrombosis/ or venous thrombosis/) (2001)
- 14  ((sinus* or sinovenous or cerebral or cavernous or sagittal venous or sagittal vein* or

cerebrovenous or cerebro-venous or sigmoid) and thrombo*).tw,kw. (62469)

- 15  intracran* thrombo*.kw. or (intracran* adj3 thrombo*).tw. (2217)
- 16  11 or 12 or 13 or 14 or 15 (134498)
- 17  10 and 16 (5558)
- 18  17 use ppez (291)
- 19  17 use cctr (53)
- 20  apixaban.mp. (10426)
- 21  edoxaban.mp. (3890)
- 22  Dabigatran.mp. (16687)
- 23  Rivaroxaban.mp. (16154)
- 24  (doac* or noac*).tw. (6344)
- 25  ((direct oral or novel) adj3 (anticoagul* or anti coagulat*)).tw. (7212)
- 26  exp *Factor Xa Inhibitors/ (9333)
- 27  Factor Xa Inhibit*.tw. (5834)
- 28  exp *thrombin inhibitor/ or thrombin* inhibit*.tw. (25389)
- 29  or/20-28 (53552)
- 30  exp cerebral sinus thrombosis/ or *occlusive cerebrovascular disease/ (11639)
- 31  ((sinus* or sinovenous or cerebral or cavernous or sagittal venous or sagittal vein* or

cerebrovenous or cerebro-venous or sigmoid) and thrombo*).tw. (60367)

- 32  (intracran* adj3 thrombo*).tw. (2149)
- 33  cvt.tw. (3510)
- 34  or/30-33 (67594)
- 35  29 and 34 (955)
- 36  35 use emczd (681)
- 37  conference abstract.pt. (2760616)
- **38  36 and 37 (223) Embase Conferences**
- 39  36 not 38 (458)
- 40  18 or 19 or 39 (802)
- 41  remove duplicates from 40 (531)
- **42  41 use ppez (255) Medline**
- **43  41 use emczd (253) Embase**
- **44  41 use cctr (23) Cochrane**
